# Automatic selection of coordinate systems for learning relative and absolute spatial concepts

**DOI:** 10.3389/frobt.2022.904751

**Published:** 2022-08-12

**Authors:** Rikunari Sagara, Ryo Taguchi, Akira Taniguchi, Tadahiro Taniguchi

**Affiliations:** ^1^ Taguchi Laboratory, Department of Computer Science, Nagoya Institute of Technology, Nagoya, Japan; ^2^ Emergent Systems Laboratory, College of Information Science and Engineering, Ritsumeikan University, Kyoto, Japan

**Keywords:** spatial concept acquisition, relative concept acquisition, coordinate system selection, lexical acquisition, bayesian nonparametrics

## Abstract

Robots employed in homes and offices need to adaptively learn spatial concepts using user utterances. To learn and represent spatial concepts, the robot must estimate the coordinate system used by humans. For example, to represent spatial concept “left,” which is one of the *relative spatial concepts* (defined as a spatial concept depending on the object’s location), humans use a coordinate system based on the direction of a reference object. As another example, to represent spatial concept “living room,” which is one of the *absolute spatial concepts* (defined as a spatial concept that does not depend on the object’s location), humans use a coordinate system where a point on a map constitutes the origin. Because humans use these concepts in daily life, it is important for the robot to understand the spatial concepts in different coordinate systems. However, it is difficult for robots to learn these spatial concepts because humans do not clarify the coordinate system. Therefore, we propose a method (RASCAM) that enables a robot to simultaneously estimate the coordinate system and spatial concept. The proposed method is based on ReSCAM+O, which is a learning method for relative spatial concepts based on a probabilistic model. The proposed method introduces a latent variable that represents a coordinate system for simultaneous learning. This method can simultaneously estimate three types of unspecified information: coordinate systems, reference objects, and the relationship between concepts and words. No other method can estimate all these three types. Experiments using three different coordinate systems demonstrate that the proposed method can learn both relative and absolute spatial concepts while accurately selecting the coordinate system. The proposed approach can be beneficial for service robots to flexibly understand a new environment through the interactions with humans.

## 1 Introduction

**FIGURE 1:**
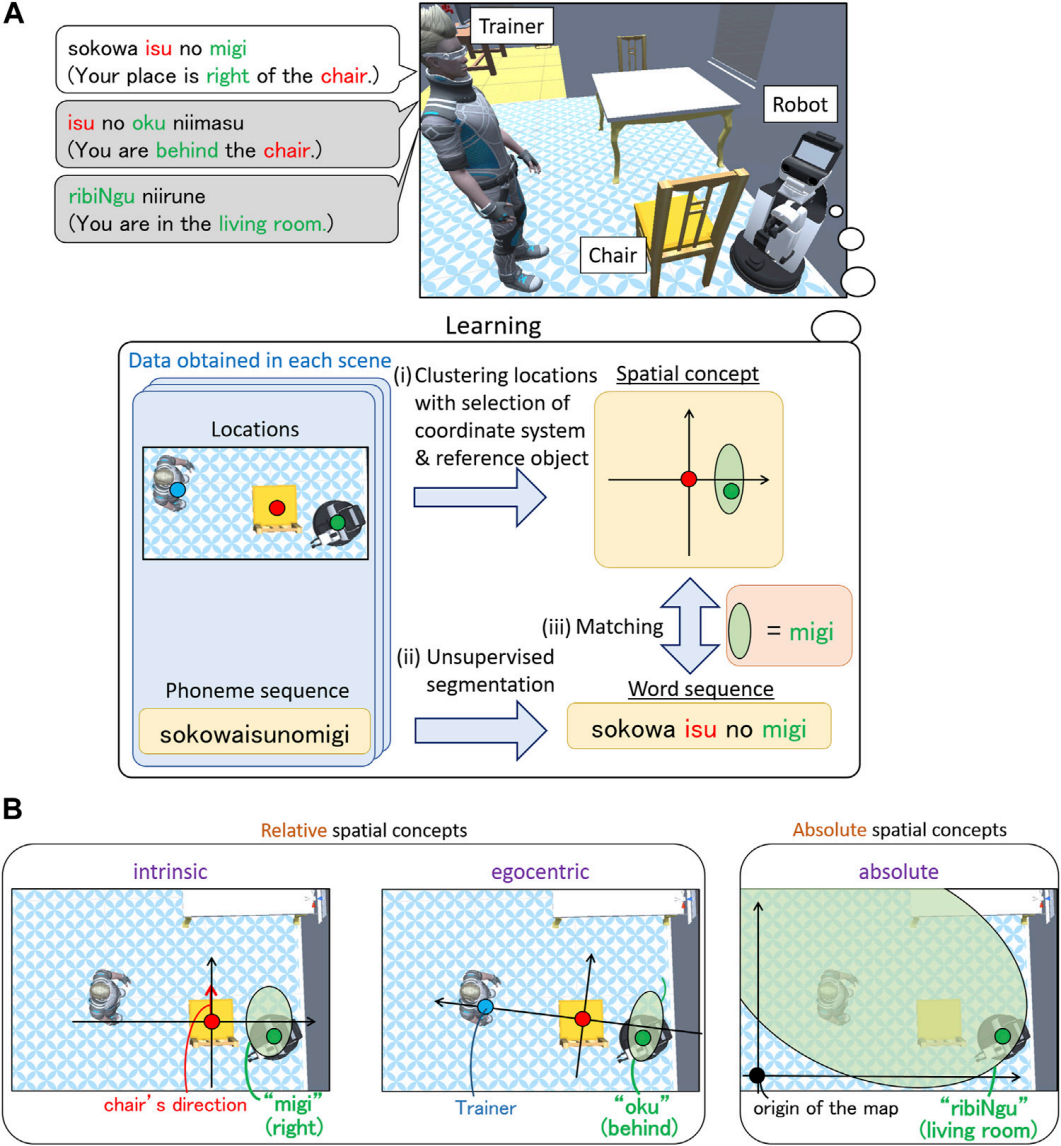
Schematic of the learning spatial concepts used in our study. **(A)** Interaction scene. A trainer teaches a robot its location using an utterance. The trainer teaches “migi” (right) in this scene. The trainer may use different concepts to represent the location as indicated by the gray speech balloons. Using the obtained locations and phoneme sequences, our method performs the following operations: i) clustering locations with the selection of coordinate systems and reference objects, ii) unsupervised segmentation of utterances, and iii) matching between a spatial concept and a word. **(B)** Coordinate systems used in our study. The trainer may use an absolute spatial concept in the absolute coordinate system or a relative spatial concept in the intrinsic or egocentric coordinate system.

Robots that support human activities in homes and offices should be able to learn spatial concepts adaptively using user utterances. Because humans use spatial concepts in multiple coordinate systems daily (Clark, 1973), it is desirable for a robot to understand the coordinate systems for learning these spatial concepts. Consider a scene in which the robot learns spatial concepts using the utterances of a trainer, as shown in Figure 1A. The trainer uses two types of spatial concepts: *relative spatial concepts*, which depend on the object’s location (e.g., front and right), and *absolute spatial concepts*, which are independent of the object’s location (e.g., kitchen and corridor). To teach the relative spatial concept “left,” the trainer uses a coordinate system based on the direction of the reference object (defined as an *intrinsic* coordinate system), as shown in Figure 1B. In contrast, to teach the relative spatial concept “behind,” the trainer uses a coordinate system based on the spatial relationship between the trainer and the object (defined as an *egocentric* coordinate system). In addition, to teach the absolute spatial concept “living room,” which does not depend on object locations, the trainer uses a coordinate system whose origin is a point on a map (defined as an *absolute* coordinate system). In general, humans do not specify the coordinate system in everyday life. Therefore, the robot must select an unspecified coordinate system to learn the spatial concepts.

Studies have been conducted on learning concepts by selecting the coordinate systems (Sugiura et al., 2011; Gu et al., 2016). Gu et al. proposed a method for learning relative spatial concepts in intrinsic and egocentric coordinate systems while estimating the coordinate systems and reference objects used by the trainer (Gu et al., 2016). However, this method cannot learn absolute spatial concepts. Robots are expected to learn both relative and absolute spatial concepts used by humans. However, a method that can learn both concepts simultaneously has not yet been developed. Further, Gu’s method cannot learn concepts using spoken utterances because the trainer utters only one word, e.g., “left,” to teach the concept. To learn concepts using utterances, for example, “Your place is left of the chair,” the robot needs to estimate the relationship between the concepts and words. Studies have been conducted on learning spatial concepts using utterances as a lexical acquisition task (Taniguchi et al., 2017, 2020a, 2020b; Sagara et al., 2022). Taniguchi et al. proposed SpCoSLAM, a learning method for absolute spatial concepts (Taniguchi et al., 2017; 2020a). This method addresses spoken utterances by learning the relationships between concepts and words obtained by unsupervised word segmentation. The acquired lexicon and spatial concepts can also be used for navigation tasks (Taniguchi et al., 2020b). In addition, Sagara et al. proposed ReSCAM+O, a learning method for relative spatial concepts using user utterances (Sagara et al., 2022). However, these methods cannot learn the spatial concepts in multiple coordinate systems. Here, we propose a method in which a robot learns these spatial concepts while estimating the coordinate system using word sequences by extending the ReSCAM+O learning method. Table 1 shows a comparison of the spatial concept learning methods. The proposed method can estimate the following unspecified elements: coordinate systems, reference objects, and the relationship between concepts and words. This method can learn both relative and absolute spatial concepts, which is not possible using any other method. In addition, the proposed method can learn concepts using word sequences by estimating the relationship between concepts and words. We performed experiments to demonstrate the aforementioned qualities of the proposed method.

**TABLE 1:** Comparison of the learning methods of spatial concepts.

	Learning spatial concepts			Learning relationship between concepts and words
	Absolute	Relative		
		Selection of References objects	Selection of coordinate systems	
Gu et al. (2016)		✓	✓	
Taniguchi et al. (2017)	✓			✓
Sagara et al. (2022)		✓		✓
Proposed method	✓	✓	✓	✓

Here, we describe the task settings used in this study. An interaction scene in the task setting of this study is illustrated in Figure 1A. The trainer and robot are in the scene, as well as the candidate reference objects. All the objects have their own direction. The trainer teaches the robot what its location is called by uttering words^1^. The trainer uses relative or absolute spatial concepts represented in an intrinsic, egocentric, or absolute coordinate system. When teaching relative spatial concepts, the trainer selects an object as the reference object among the candidate reference objects. Such teaching is iterated several times by changing the locations of the trainer and robot. The robot does not know the reference objects, coordinate system used by the trainer, and the boundaries of the words because it has no pre-existing lexicon. The robot has an acoustic model and a language model of Japanese syllables as its initial knowledge and can recognize an utterance as a phoneme sequence. In addition, the robot can recognize each object as an object category. The robot learns spatial concepts and the words representing them while estimating the reference object, coordinate system, and relationship between the concepts and words in each scene.

The main contributions of this paper are as follows:

- We propose a novel method that can learn both relative and absolute spatial concepts without any prior distinctions.
- We show that our proposed method can select coordinate systems and learn spatial concepts represented in three different coordinate systems using word sequences.
- We show that our proposed method outperforms other methods that do not select coordinate systems.

The remainder of this paper is organized as follows. In Section 2, we discuss previous relevant studies. In Section 3 and Section 4, we present our previous ReSCAM+O method and our proposed method, respectively. In Section 5, the experimental results obtained using the proposed method are presented. Section 6 includes the conclusions drawn from the results of this study.

## 2 Related work

### 2.1 Simultaneous learning of concepts and words

Studies have been conducted on simultaneous learning of concepts and words. Frank et al. proposed a Bayesian model for cross-situational learning of words (Frank et al., 2008). Their model clarified the understanding of word learning, which mentions that a Bayesian model can be easily extended for joint learning with other domains. Therefore, the proposed model is based on a Bayesian model. Heath et al. proposed a learning method for lexical knowledge using robot-to-robot communication (Heath et al., 2016). They showed that this method could resolve referential uncertainty for the dimensions of space and time. Štepánová et al. suggested a method for mapping language to vision using a real-world robotic scenario (Štepánová et al., 2018). This method could robustly find the mapping between language and vision. However, none of these methods can learn the phoneme sequences of unknown words in utterances because word segmentation is not performed.

Studies have also been conducted on learning unknown words using unsupervised word segmentation. Synnaeve et al. proposed word segmentation methods using a nonlinguistic context (Synnaeve et al., 2014). The results showed that the model produced better segmentation results than its context-oblivious counterparts. However, this method requires labels for context annotations. Incorrect labels can be estimated when a robot learns concepts using sensory information. In our model, labels are estimated using sensory information as well as word sequences to perform mutual complementation of the ambiguities. Araki et al. suggested a method for learning object concepts and word meanings using multimodal information and spoken sentences (Araki et al., 2012). Similarly, Nakamura et al. proposed a mutual learning method based on integrating the learning of object concepts with a language model (Nakamura et al., 2014). In these methods, spoken sentences are segmented using an unsupervised morphological analyzer based on a nested Pitman-Yor language model (NPYLM) (Mochihashi et al., 2009). However, using NPYLM, the word boundaries were not estimated correctly when the recognized phoneme sequences contained errors. To solve this problem, Taniguchi et al. proposed SpCoA++ (Taniguchi et al., 2018) using Neubig’s unsupervised word segmentation method (Neubig et al., 2012), which uses speech recognition lattices. Our previous method for relative spatial concepts also used the segmentation method to solve this problem (Sagara et al., 2022).

### 2.2 Learning relative concepts

The learning of related concepts has also been studied. Tellex et al. proposed a probabilistic learning framework for spatial concepts (spatial relationships) using natural sentences (Tellex et al., 2011). A robot trained by their method can learn and use word meanings in real-world tasks. Aly and Taniguchi presented a learning method for spatial concepts which represents spatial relationship between objects in a tabletop scene (Aly and Taniguchi, 2018). This method enables a robot to perform actions on objects using a sentence, for example, “Raise the red bottle near the box.” Sagara et al. suggested ReSCAM+O, a learning method for relative spatial concepts using user utterances (Sagara et al., 2022). However, these methods cannot learn concepts using two or more coordinate systems. Studies have been conducted on multiple coordinate systems for spatial concepts in cognitive science (Landau and Jackendoff, 1993; Gapp, 1994; Imai et al., 1999). In artificial intelligence, there are studies on learning spatial/motion concepts in two or more coordinate systems. Iwata et al. proposed a learning method for motion relative to a reference point (Iwata et al., 2018). Coordinate systems were selected during the learning process. However, this method cannot consider multiple coordinate systems for each object. Spranger et al. suggested a method for learning relative spatial concepts similar to our study (Spranger, 2013, 2015). This method could learn relative spatial concepts in different coordinate systems. However, they did not consider several candidate reference objects.

Studies have been conducted on learning concepts while estimating the coordinate systems as well as reference objects. Sugiura et al. proposed a learning method of relative spatial moving concepts by estimating both reference objects and coordinate systems using an expectation-maximization (EM) algorithm (Sugiura et al., 2011). Gu et al. proposed a method for learning relative spatial concepts in different coordinate systems using an EM algorithm (Gu et al., 2016). However, in these studies, the concepts cannot be learned using human utterances because the robot must know in advance the concept being taught. The proposed method can learn concepts using user utterances by estimating all the reference points, coordinate systems, and concepts being taught.

## 3 Previous method: ReSCAM+O

This section describes the spatial concept acquisition method using reference object clues (ReSCAM+O) on which the proposed method described in Section 4 is based.

### 3.1 Overview

**FIGURE 2:**
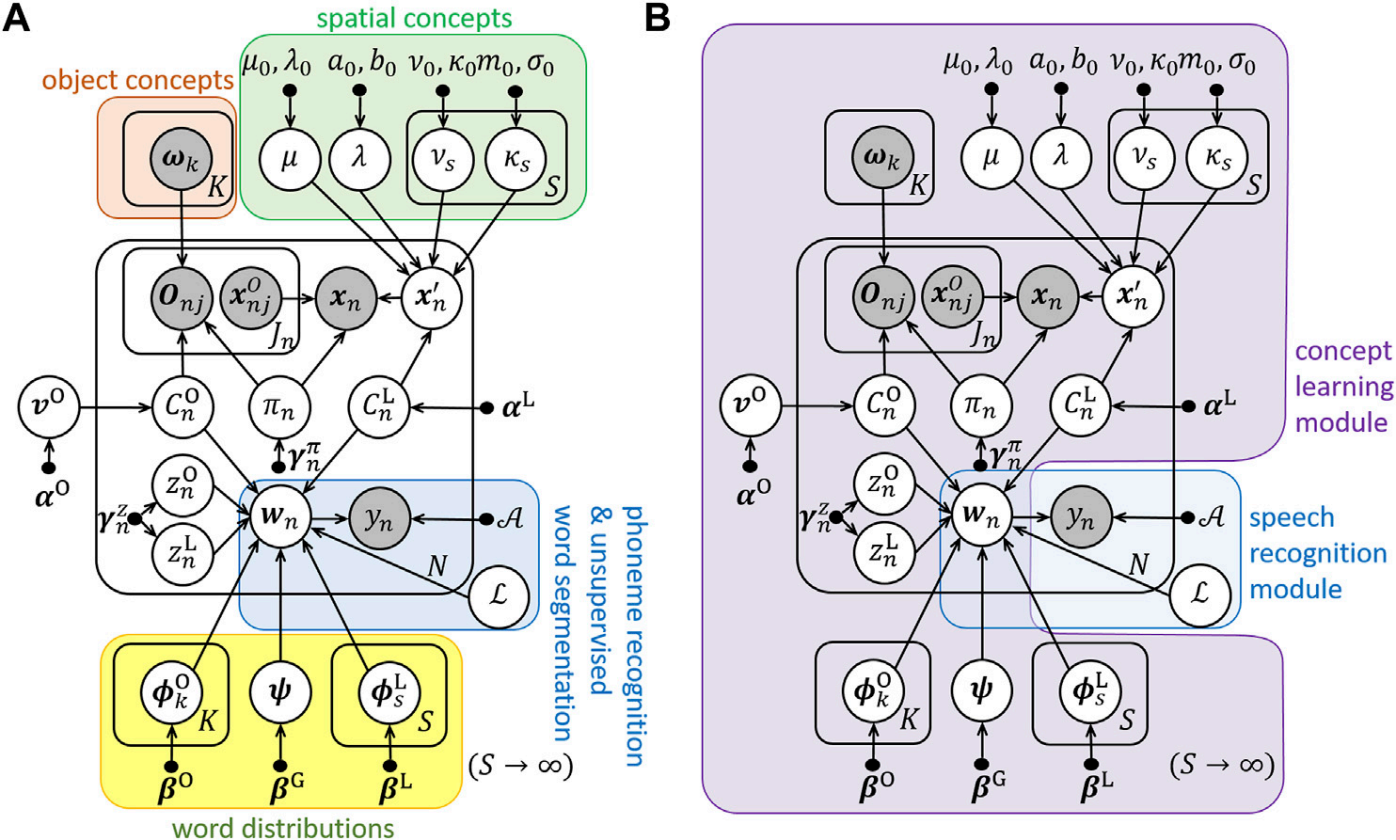
Graphical models of ReSCAM+O. **(A)** Functions of variables. **(B)** Modules.

ReSCAM+O enables robots to segment words accurately and learn relative spatial concepts. This method is based on a probabilistic model. Figure 2A shows a graphical model of ReSCAM+O and Table 2 lists the variables used in ReSCAM+O. As shown in Figure 2B, the probabilistic model comprises the concept learning module and speech recognition module. The details of the ReSCAM+O generation process are described in (Sagara et al., 2022). This method can learn relative spatial concepts as distributions by estimating the reference object in each scene. The number of concepts during learning are estimated using the Chinese restaurant process (CRP) (Aldous, 1985). In addition, it learns novel words using an unsupervised word segmentation method (latticelm) (Neubig et al., 2012), class n-gram, and the selection of segmentation candidates using mutual information. Furthermore, it can be used to learn the relationship between concepts and words. The method learns them simultaneously to compensate for the uncertainty of the inputs.

**TABLE 2:** Variables of ReSCAM+O.

$C_{n}^{\text{L}}$	Index of spatial concepts
$C_{n}^{\text{O}}$	Index of object categories
$\pi_{n}$	Index of References objects
$x_{n}$	Absolute location of the trainer
$x_{n}^{\prime }$	Location in a selected coordinate system
$x_{nj}^{O}$	Location of candidate References objects
μ	Mean of distance
λ	Precision of distance
$\nu_{s}$	Mean angle of relative spatial concepts
$\kappa_{s}$	Concentration of relative spatial concepts
$\omega_{k}$	Parameter of distribution of object recognition result
$v^{O}$	Parameter of prior of index of object categories
$O_{nj}$	Object recognition result of each candidate References object
$w_{n}$	Word sequences
$\phi_{s}^{\text{L}}$	Parameter of word distribution of spatial concepts
$\phi_{k}^{\text{O}}$	Parameter of word distribution of object categories
ψ	Parameter of word distribution of concept-independent words
$z_{n}^{\text{L}}$	Index of location words
$z_{n}^{O}$	Index of object words
$y_{n}$	Utterance
A	Acoustic model
ℒ	Language model
$\mu_{0},\;\lambda_{0},\;a_{0},\;b_{0},\nu_{0},\;\kappa_{0},\;m_{0},\;\sigma_{0},e,\alpha^{\text{O}},\alpha^{\text{L}},\beta^{\text{L}},\beta^{\text{O}},\;\beta^{\text{G}},\gamma_{n}^{\pi },\gamma_{n}^{z}$	Hyperparameters
N	Number of scenes
S	Number of spatial concepts
K	Number of object categories
$J_{n}$	Number of candidate References objects

### 3.2 Probabilistic generative model

The relative location $x_{n}^{\prime }$ is generated as follows.

$$x_{n}^{\prime }\;∼\text{ N}\left(l_{n}|\mu ,\lambda^{-1}\right)\text{vM}\left(\theta_{n}|\nu_{C_{n}^{\text{L}}},\kappa_{C_{n}^{\text{L}}}\right)$$ (1)

where $l_{n}$ denotes the distance between a reference object and trainer, and $\theta_{n}$ denotes the angle between a line that passes through the reference object and trainer and a line that passes through the reference object and robot. The distance $l_{n}$ is generated using a normal distribution N(⋅) , and the angle $\theta_{n}$ is generated using the von Mises distribution vM(⋅), which can represent angles or directions.

The absolute location of the trainer $x_{n}$ is generated using the relative location $x_{n}^{\prime }$ as follows:

$$x_{n}=x_{n\pi_{n}}^{O}+R\left(f_{n\pi_{n}}^{O}\right)x_{n}^{\prime }$$ (2)

where R(θ) denotes the rotation matrix of angle θ and $f_{n\pi_{n}}^{O}$ denotes the direction of the reference object.

A word sequence $w_{n}$ is generated using an approximation through unigram rescaling (Gildea and Hofmann, 1999) to connect the concept learning module and speech recognition module, as follows:

$$\begin{array}{l} w_{n}\;∼\;p\left(w_{n}|\phi^{\text{L}},\phi^{\text{O}},\psi ,C_{n}^{\text{L}},\;C_{n}^{\text{O}},z_{n}^{\text{L}},z_{n}^{\text{O}},ℒ\right)\\ \overset{\text{UR}}{\approx }p\left(w_{n}\text{|}ℒ\right)\underset{i}{\prod }\frac{p\left(w_{ni}|\phi^{\text{L}},\phi^{\text{O}},\psi ,C_{n}^{\text{L}},C_{n}^{\text{O}},z_{n}^{\text{L}},z_{n}^{\text{O}}\right)}{p\left(w_{ni}\right)} \end{array}$$ (3)

where $\phi^{\text{L}}=\left\{\phi_{1}^{\text{L}},\ldots ,\phi_{S}^{\text{L}}\right\}$ , $\phi^{O}=\left\{\phi_{1}^{\text{O}},\ldots ,\phi_{K}^{\text{O}}\right\}$ ; $\overset{\text{UR}}{\approx }$ denotes an approximation using unigram rescaling; $p\left(w_{ni}|\phi^{\text{L}},\phi^{\text{O}},\psi ,C_{n}^{\text{L}},C_{n}^{\text{O}},z_{n}^{\text{L}},z_{n}^{\text{O}}\right)$ denotes the prior probability of $w_{ni}$ , the i− th word of word sequence $w_{n}$ . This is calculated as follows:

$$p\left(w_{\mathrm{ni}}|\phi^{L},\varphi^{O}\mathrm{,\psi ,}C_{n}^{L},C_{n}^{O},z_{n}^{L},z_{n}^{O}\right)=\{\begin{array}{c} \mathrm{Mult}\left(w_{\mathrm{ni}}|\phi_{C_{n}^{L}}^{L}\right)\;\left(z_{n}^{L}=i\right)\\ \mathrm{Mult}\left(w_{\mathrm{ni}}|\phi_{C_{n}^{O}}^{O}\right)\;\left(z_{n}^{O}=i\right)\\ \mathrm{Mult}\left(w_{\mathrm{ni}}\mathrm{| \psi}\right)\;\left(\mathrm{otherwise}\right) \end{array}$$ (4)

where $\phi_{s}^{\text{L}},\;\phi_{k}^{\text{O}}$ denote the word distributions of a spatial concept and an object, respectively, ψ denotes the word distribution of concept-independent words, and $z_{n}^{\text{L}},z_{n}^{\text{O}}$ denote the indices of words representing a spatial concept and an object, respectively. The words selected by $z_{n}^{\text{L}},z_{n}^{\text{O}}$ are defined as *location words* and *object words*, respectively. Eq. 4 indicates that each utterance is assumed to have one location word and one object word.

An object recognition result of the reference object $O_{n\pi_{n}}$ is generated as follows:

$$O_{n\pi_{n}}\;∼\text{ Mult}\left(\omega_{C_{n}^{\text{O}}}\right)$$ (5)

where $\omega_{k}$ denotes the parameter of the distribution. The object recognition result represents the probability that a candidate reference object will be classified into each object category. The object recognition result is used as a clue for estimating reference objects.

### 3.3 Parameter estimation

The estimated parameters are as follows: parameters of the spatial concepts $\mu ,\;\lambda ,\;\nu_{s},\;\kappa_{s}$ , parameters of the word distributions $\phi_{s}^{\text{L}}$ , $\phi_{k}^{\text{O}}$ , ψ , parameter of the prior for the indices of the object categories $v^{O}$ , word sequences $w_{n}$ , language model ℒ , and indices $C_{n}^{\text{L}}$ , $C_{n}^{\text{O}}$ , $\pi_{n}$ , $z_{n}^{\text{L}}$ , $z_{n}^{\text{O}}$ . The probabilistic model parameters are estimated by iterating the following four steps: (a) generating word sequences, (b) concept learning, (c) selecting a list based on mutual information, and (d) updating the language model. The parameters in the concept learning module without word sequence $w_{n}$ are estimated in (b), word sequence $w_{n}$ is estimated in (a) and (c), and language model ℒ is estimated in (d). In step (b), the parameters are estimated using the Metropolis-Hastings (M-H) method, which is a Markov-chain Monte Carlo (MCMC) method. During the iterations of the M-H method, the number of spatial concepts is also estimated using CRP.

## 4 Proposed method: Relative and absolute spatial concept acquisition method

### 4.1 Probabilistic generative model

**FIGURE 3:**
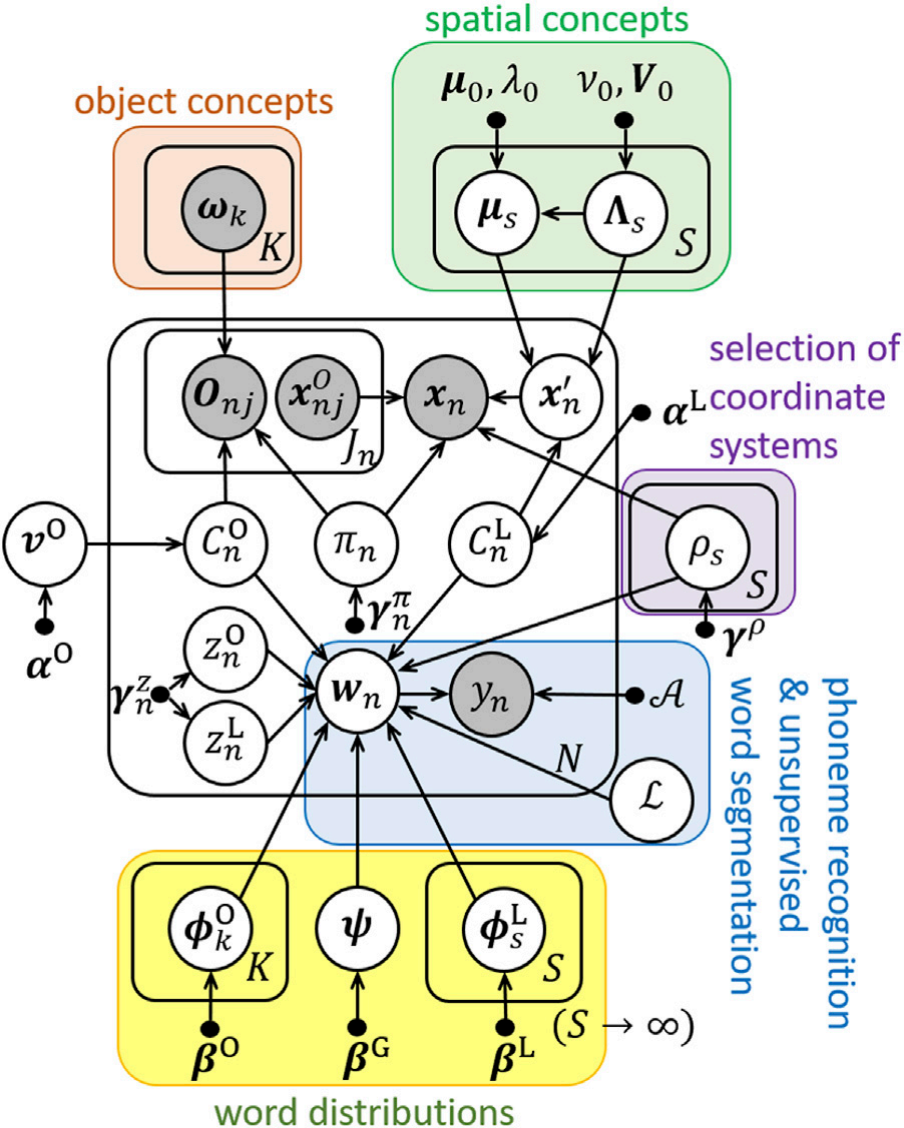
Graphical model of RASCAM. A new variable $\rho_{s}$ is added.

The proposed method RASCAM enables robots to learn both relative and absolute spatial concepts without any prior distinctions. In the proposed method, the concept learning module of ReSCAM+O is improved. Figure 3 shows a graphical model of the proposed method and Table 3 lists the new variables used in the proposed method. We added a new variable $\rho_{s}\in \left\{\text{ABS},\text{ REL}\_\text{INTRINSIC},\text{ REL}\_\text{EGOCENTRIC}\right\}$ , which denotes the coordinate system of concept s . When $\rho_{s}=\text{ABS}$ , concept s is an absolute spatial concept in an absolute coordinate system. When $\rho_{s}=\text{REL}\_\text{INTRINSIC}$ , concept s is a relative spatial concept in an intrinsic coordinate system. When $\;\rho_{s}=\text{REL}\_\text{EGOCENTRIC}$ , concept s is a relative spatial concept in an egocentric coordinate system. The location in the coordinate system $\rho_{C_{n}^{L}}$ is denoted as $x_{n}^{\prime }$ , which is generated by the distribution of the concept $C_{n}^{\text{L}}$ , as shown in (6).

$$x_{n}^{\prime }\;∼\;N\left(\mu_{C_{n}^{\text{L}}},\Lambda_{C_{n}^{\text{L}}}\right)$$ (6)

where $\mu_{C_{n}^{\text{L}}},\Lambda_{C_{n}^{\text{L}}}$ denote the parameters of the distribution, N(⋅) denotes a normal distribution, and $C_{n}^{\text{L}}$ denotes an index of a spatial concept uttered in scene n . In the previous method, a relative spatial concept was represented by an angle distribution and a distance distribution. However, these distributions cannot represent absolute spatial concepts. In the proposed method, both relative and absolute spatial concepts are represented as normal distributions to easily analyze the results.

**TABLE 3:** New variables of RASCAM.

$\mu_{s}$	Mean vector of spatial concepts
$\Lambda_{s}$	Precision matrix of spatial concepts
$\rho_{s}$	Coordinate system of spatial concepts
$\gamma^{\rho }$	Hyperparameter

Location $x_{n}^{\prime }$ is transformed into an absolute coordinate system, as shown in (7).

$$x_{n}=\{\begin{array}{c} x_{n}^{\prime }\;\;\;\;\;\;\;\;\rho_{C_{n}^{\text{L}}}=\mathrm{ABS}\\ x_{n\pi_{n}}^{O}+R\left(f_{n\pi_{n}}^{O}\right)x_{n}^{\prime }\;\;\;\;\;\;\;\;\rho_{C_{n}^{\text{L}}}=\mathrm{REL}\_\mathrm{INTRINSIC}\\ x_{n\pi_{n}}^{O}+R\left(f_{n\pi_{n}}^{Tr}\right)x_{n}^{\prime }\;\;\;\;\;\;\;\;\rho_{C_{n}^{\text{L}}}=\mathrm{REL}\_\mathrm{EGOCENTRIC} \end{array}$$ (7)

where $x_{n\pi_{n}}^{O}$ denotes the location of the object, R(θ) denotes the rotation matrix of angle θ , $f_{n\pi_{n}}^{O}$ denotes the direction of the reference object, and $f_{n\pi_{n}}^{Tr}$ denotes the direction from the reference object to the trainer. Unlike (2), different transformations are used depending on $\rho_{C_{n}^{L}}$ . Parameters $\mu_{s},\Lambda_{s}$ are generated from their conjugate priors: a normal distribution and Wishart distribution, respectively.

Word $w_{ni}$ , which is the i - th word of word sequence $w_{n}$ , is generated as shown in (8).

$$w_{ni}∼\{\begin{array}{c} \mathrm{Mult}\left(w_{ni}\text{|}\phi_{C_{n}^{\text{L}}}^{\text{L}}\right)\;\;\;\;\left(z_{n}^{\text{L}}=i\right)\\ \mathrm{Mult}\left(w_{ni}\text{|}\phi_{C_{n}^{\text{O}}}^{\text{O}}\right)\;\;\;\;\left(z_{n}^{\text{O}}=i\;\;\text{and}\;\;\rho_{C_{n}^{\text{L}}}\neq \text{ABS}\right)\\ \mathrm{Mult}\left(w_{ni}\text{|}\;\psi \right)\;\;\;\;\;\;\left(\text{otherwise}\right) \end{array}.$$ (8)

This shows that utterances representing relative spatial concepts are assumed to have a location word and an object word, and utterances representing absolute spatial concepts are assumed to have a location word but no object word.

### 4.2 Parameter estimation

For parameter estimation, the difference between the learning algorithm and ReSCAM+O is described. The new parameter $\rho_{s}$ is estimated using the M-H method in step (b) similar to the other parameters in the concept learning module. In ReSCAM+O, steps (a–d) are repeated, as described in Section 3.3. However, steps (a), (c), and (d) cannot be directly applied to the proposed method for estimating the word sequences and language model. Because it is possible to segment words to a certain extent without using these improvements, step (b) alone is performed using the word sequences obtained through unsupervised word segmentation instead of repeating steps (a–d).

## 5 Experiments

### 5.1 Conditions

To demonstrate the advantages of learning concepts while selecting the coordinate system, we compared the learning results obtained using the following five methods:

A) learning only in the absolute coordinate system,
B) learning only in the intrinsic coordinate system,
C) learning only in the egocentric coordinate system,
D) proposed method $\left(\lambda_{0}^{\text{R}}=0.01,\lambda_{0}^{\text{A}}=0.01\right)$ , and
E) proposed method $\left(\lambda_{0}^{\text{R}}=1.00,\lambda_{0}^{\text{A}}=0.01\right)$ .

Methods (B) and (C) are our previous methods ReSCAM+O (Sagara et al., 2022) in which the distributions of the spatial concepts are replaced by two-dimensional normal distributions. Although the baseline can be calculated using another method such as SpCoA that does not estimate coordinate systems, applying the other method to a task for which it was not designed would unreasonably lower the values obtained in the evaluation. Therefore, in this study, we evaluated the baseline performance by excluding the estimation of the coordinate systems from the proposed method.

**FIGURE 4:**
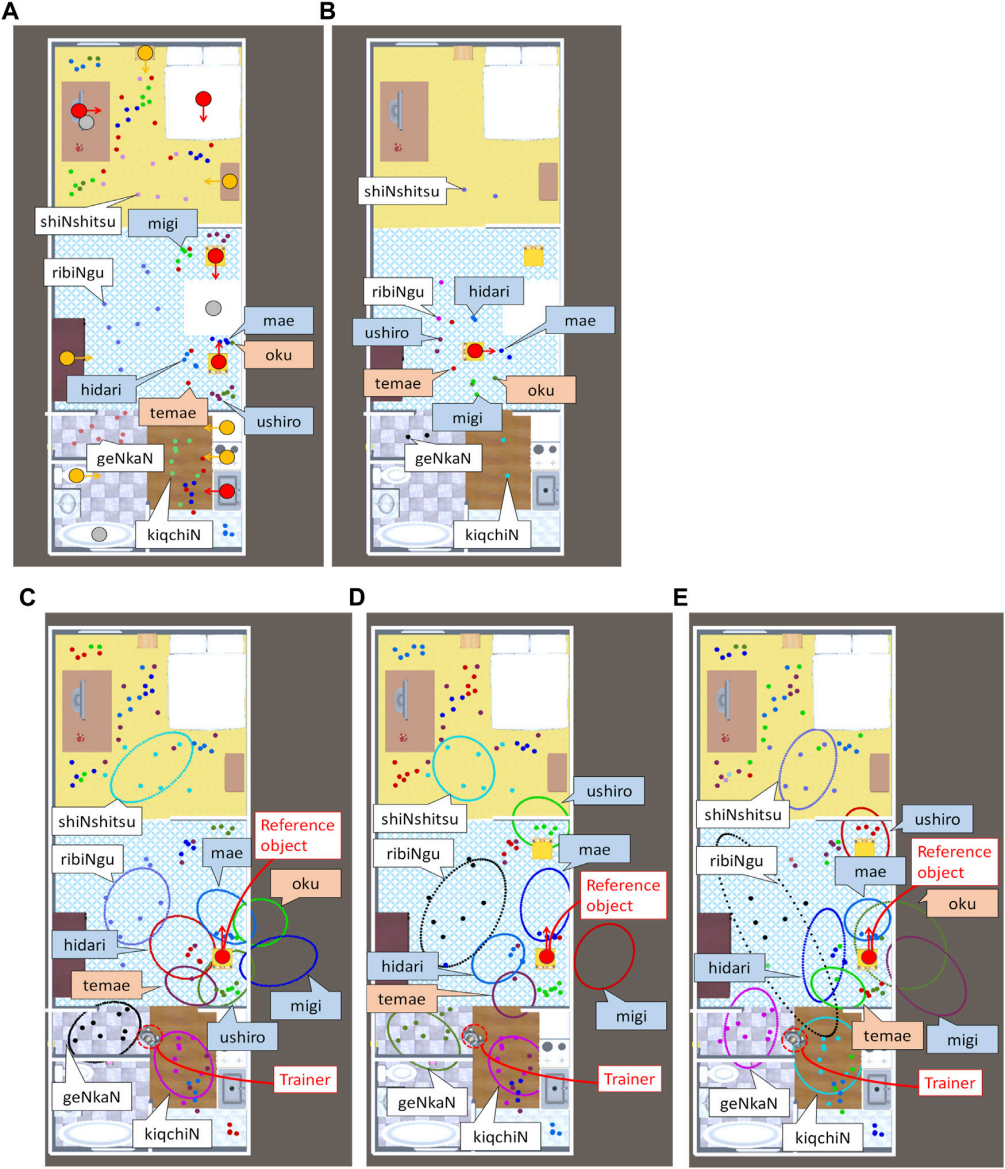
Locations taught by the trainer and spatial concepts learned in experiments with utterances. The dots represent the locations taught by the trainer. The colors of the word boxes represent the coordinate systems: absolute (white), intrinsic (blue), egocentric (red). **(A)** Training data. The red circles represent the candidate reference objects used for teaching. The orange circles represent the candidate reference objects not used for teaching. The gray circles represent nondirectional objects, which are not candidate reference objects. The arrows represent the directions of the objects. **(B)** Test data. **(C–E)** Result D-best, Result D-typical, and Result E. The is not displayed for visibility of the spatial concepts. The ellipses represent the normal distribution of the absolute spatial concepts and relative spatial concepts where a chair at the bottom is used as a reference object. The ellipses do not represent the boundaries of the concepts. The locations are associated with concepts that have the same color as the dot. The spatial concepts in the egocentric coordinate system are drawn using the trainer’s location.

A study on the spatial concept acquisition task for robots (Taniguchi et al., 2020a), SIGVerse, (Inamura and Mizuchi, 2021) used an architecture that connects Unity and ROS. As in their study, we used a virtual home environment^2^ in Unity. The trainer and robot in the environment were controlled using a keyboard. The robot can detect candidate reference objects in the environment and recognize their directions. We used 12 directed objects as candidate reference objects. Among the objects, we used four as the reference objects. We assumed that the object recognition had no errors. The objects were classified into ten categories. We taught the robot’s location in 104 scenes using spatial concepts^2^,^3^. Figure 4A shows the locations of the robots taught by the trainer. We taught four absolute spatial concepts, four relative spatial concepts represented in an intrinsic coordinate system, and two relative spatial concepts expressed in an egocentric coordinate system. In this experiment, to focus on whether spatial concepts can be learned while selecting coordinate systems, we used the correct word segmentation results of user utterances as an input^4^. Therefore, learning was performed using only (b) concept learning, as shown in Section 4.2. The experiment was performed ten times by changing the initial values of the parameters. The hyperparameter values were set as follows: $\mu_{0}={\left(0.0,\;0.0\right)}^{T},\nu_{0}=3.0,V_{0}=I$ , $\alpha^{\text{L}}=1.0$ , $\alpha^{\text{O}}={\left(1.0,\ldots ,1.0\right)}^{T}$ , $\beta^{\text{L}}={\left(0.1,\ldots ,0.1\right)}^{T}$ , $\beta^{\text{O}}={\left(0.1,\ldots ,0.1\right)}^{T}$ , $\beta^{\text{G}}={\left(0.1,\ldots ,0.1\right)}^{T}$ , $\;\gamma_{n}^{\pi }∼{\left(1.0,\ldots ,1.0\right)}^{T}$ , $\gamma_{n}^{z}∼{\left(1.0,\ldots ,1.0\right)}^{T}$ , $\gamma^{\rho }∼{\left(1.0,\ldots ,1.0\right)}^{T}$ .

In addition, for the proposed method, experiments were conducted by setting $\lambda_{0}$ , which indicates the distance between the center of the distribution and origin of the coordinate system, to two values. First, to prevent the relative and absolute spatial concepts from being distinguished by using the distance from the origin of the coordinate system, the hyperparameters for both the relative and absolute spatial concepts $\lambda_{0}^{\text{R}},\lambda_{0}^{\text{A}}$ were set to 0.01 in proposed method (D). As the actual relative spatial concepts are rarely taught far away from the reference object, in method (E), the hyperparameters $\lambda_{0}^{\text{R}},\lambda_{0}^{\text{A}}$ were set to 1.00, 0.01, respectively. This facilitates distinction between relative and absolute spatial concepts. For the other methods, hyperparameters $\lambda_{0}^{\text{R}},\lambda_{0}^{\text{A}}$ were set to 0.01 as in method (D). The number of learning iterations was 20,000 and the value of the final iteration was used as the result. To calculate the evaluation metric WAR described in Section 5.2, we used the test data of the locations in 20 scenes in which a chair’s location was moved, as shown in Figure 4B.

### 5.2 Metrics

The following evaluation metrics are used to evaluate whether each spatial concept is learned while distinguishing the coordinate system, and whether the location and word can be mutually estimated. CAR, RAR, and ARI evaluate the results using the training data, whereas WAR evaluates the results using test data.

- Coordinate system accuracy rate (CAR) Percentage of scenes where the coordinate system $\rho_{C_{n}^{\text{L}}}$ of the selected spatial concept $C_{n}^{\text{L}}$ is the correct value for the training data. We evaluated whether the coordinate system could be estimated accurately.
- Reference object accuracy rate (RAR)Percentage of scenes in which the reference object $\pi_{n}$ could be correctly estimated among the scenes that were correctly estimated as the relative spatial concept for the training data.
- Estimation accuracy rate of the spatial concepts (ARI). For the training data, the estimation accuracy of the index of the spatial concept $C_{n}^{\text{L}}$ . The adjusted Rand index (ARI) (Hubert and Arabie, 1985), which represents the similarity between two data clustering, evaluates the similarity between the correct and estimated values. The ARI was 1.0, when the estimated values were exactly the same as the correct values.
- Word Accuracy Rate (WAR)Percentage at which the location word w estimated from the test data of locations $x_{n}^{\text{test}}$ matches the correct answer using the learned parameters. This metric assumes that the task of the robot is to answer the name of the requested location. This metric evaluates whether the spatial concepts and word distributions are learned correctly. The robot may have several candidate words to answer by changing the coordinate system and reference objects. The preferred coordinate system and reference objects are unclear. However, as the focus was on learning the concepts in each coordinate system, this problem was not addressed in this study. Using coordinate system $\rho^{\text{test}}$ and reference object $\pi^{\text{test}}$ , we evaluated whether the robot could estimate the word correctly. The location word w was estimated using the following equation:

$$w=\underset{w}{\mathrm{argmax}}p\left(w\text{|}x_{n}^{\mathrm{test}},\Theta ,\;\pi^{\mathrm{test}},\rho^{\mathrm{test}}\right)$$

$$=\underset{w}{\mathrm{argmax}}\underset{s:\rho_{s}=\rho^{\mathrm{test}}}{\sum }p\left(w\text{|}\phi_{s}^{L}\right)p\left(C_{n}^{L}=s\text{|}\alpha^{L}\right)p\left(x_{n}^{\mathrm{test}}\text{|}\mu ,\Lambda ,\;\pi_{n}=\pi^{\mathrm{test}},C_{n}^{L}=s,\rho_{s}\right)$$ (9)

### 5.3 Results and discussion

In this section, we discuss whether these methods can learn spatial concepts in different coordinate systems. Table 4 lists the averages of the evaluation values.

**TABLE 4:** Evaluation results.

Methods	CAR	RAR	ARI	WAR
(A) learning only in the absolute sys	N/A	N/A	0.283^a^	0.360^a^
(B) learning only in the intrinsic sys	N/A	0.563^a^	0.426^a^	0.230^a^
(C) learning only in the egocentric sys	N/A	0.925^b^	0.422^a^	0.200^a^
(D) proposed method ( $\lambda_{0}^{\text{R}}=0.01$ )	0.883	0.782	0.832	0.800
(E) proposed method ( $\lambda_{0}^{\text{R}}=1.00$ )	**0.964**	**0.984**	**0.945**	**0.960**

a Significantly lower at 0.05 level in comparison with the method (D).

b Significantly higher at 0.05 level in comparison with the method (D).

The bold values indicate the highest values.

#### 5.3.1 Evaluation of the learning results of the proposed method

We evaluated the results of proposed methods D and E. First, we considered the best learning result example (result D-best) of proposed method (D), as shown in Figure 4C. The figure shows that the distributions of the relative as well as absolute spatial concepts are successfully learned. It also shows that the coordinate systems and the relationship between the concepts and words are correctly estimated. In result D-best, the evaluation values are CAR = 0.990, RAR = 1.000, ARI = 0.976, and WAR = 1.000. This result shows that the location data are clustered ideally if the reference objects and coordinate systems are correctly estimated. In contrast, we focus on the typical learning result (called Result D-typical) of proposed method (D) shown in Figure 4D. In Result D-typical, the evaluation values are close to the average: CAR = 0.903, RAR = 0.819, ARI = 0.855, and WAR = 0.800. The figure shows that the coordinate systems and relationships between concepts and words of the learned concepts are estimated correctly. However, concept “oku” (behind) was not learned. In addition, concept “ushiro” (back) was erroneously learned as a distribution far in front of a chair. This is caused by a combination of the following two conditions: i) objects in the same category are placed facing each other and ii) when teaching “ushiro,” the reference objects are the same (chairs). In case i), clustering is also possible by learning as a distribution far in front of the reference object using the intrinsic coordinate system. In case ii), even if another chair is selected as the reference object, the likelihood does not decline because the object categories are the same. The learned concept “ushiro” can be used only when the conditions do not change. WAR is low because the position of the chair is changed. Concept “ushiro” is more difficult to learn than the other concepts in a home environment because most furniture are placed near the wall and face inward. It can be correctly learned by increasing the variation in the teachings, e.g., using other reference objects or moving reference objects. In another learning result of proposed method (D), the learning accuracy is reduced owing to the learning of relative spatial concepts that are extremely far from the reference object. The evaluation values are CAR = 0.452, RAR = 0.000, ARI = 0.353, and WAR = 0.400. RAR is below 0.800 in only one out of 10 cases. Consequently, although proposed method (D) can learn concepts to a certain extent, it has a problem with the learning stability.

For proposed method (E), setting $\lambda_{0}^{\text{R}}=1.00$ reduces the learning of such erroneous concepts and improves the performance. The learning results (result E) are shown in Figure 4E. For visibility, four concepts, in which only one location data point is classified, are not displayed in the figure. These concepts do not affect WAR because $p\left(C_{n}^{\text{L}}=s\text{|}\alpha^{\text{L}}\right)$ of the concept in Eq. 9 is small. Except these concepts and concept “ushiro,” the spatial concepts are learned correctly. The evaluation values are CAR = 0.952, RAR = 0.889, ARI = 0.917, and WAR = 0.900. Although RAR is the lowest in 10 trials, it is higher than the average of proposed method (D). This shows that proposed method (E) can learn spatial concepts stably when the reference objects are correctly estimated by setting $\lambda_{0}^{\text{R}}$ and $\lambda_{0}^{\text{A}}$ to ensure that the relative and absolute spatial concepts have different properties.

#### 5.3.2 Verification of the effectiveness of coordinate system selection

For RAR, ARI, and WAR, shown in Table 4, Wilcoxon rank sum tests^5^ were performed on the results of proposed method (D) and methods (A, B, C), respectively. For both ARI and WAR, proposed method (D) generates significantly higher evaluation values. On the other hand, proposed method (D) generates significantly higher RAR than method (B), and significantly lower RAR than method (C). We discuss the results of each method in the following order. Method (A) has a low ARI because the learning of the relative spatial concept fails. The position for teaching the relative spatial concept was learned as an absolute spatial concept by estimating an object word as a location word. In addition, even if the concepts and word distributions are correctly learned using only the absolute coordinate system, the versatility of the learned concept is low for the following reasons. First, they cannot respond to the changes in the location or direction of an object. In addition, they cannot respond to utterances that are newly encountered combinations of reference objects and relative spatial concepts.

Next, using method (B), the absolute spatial concepts were learned as relative spatial concepts by selecting a specific reference object. Using the learned distribution, if the reference object can be estimated correctly, the word and position can be mutually estimated. However, with this method, the absolute spatial concepts are not correctly expressed when the position or direction of the object changes. Moreover, because the teaching of the concept of the egocentric coordinate system depends on the trainer’s location, clustering cannot be performed correctly by this method, which does not consider the trainer’s location. Consequently, the WAR of this method is lower than that of the proposed method, despite the high RAR value.

Furthermore, using method (C), the RAR is higher than method (D) because method (C) does not use the intrinsic coordinate system, which causes erroneous learning results for concept “ushiro” (see section 5.3.1). However, the absolute spatial concepts are divided into several distributions because the relative location of the absolute spatial concept changes depending on the trainer’s location, unlike the case of the intrinsic coordinate system. As a result, method (C) generates low ARI and WAR.

The above discussion shows that the proposed method, which selects the coordinate systems, is excellent in learning from the teaching of concepts in different coordinate systems. The discussion implies that concepts cannot be learned without selecting coordinate systems. In addition, it is shown that the proposed method significantly outperforms the other methods.

## 6 Conclusion

This study proposed a method for learning relative and absolute spatial concepts while appropriately selecting the coordinate systems. In the proposed method, a latent variable that represented three types of coordinate systems was adopted. The coordinate system was estimated simultaneously with the spatial concept. Experiments were conducted using data that taught the spatial concepts expressed in the intrinsic, egocentric, and absolute coordinate systems. The experimental results showed that the proposed method could learn spatial concepts while selecting the coordinate system. In addition, it was demonstrated that the estimation accuracy of the spatial concept was improved by selecting the coordinate system. This enables the robot to learn the spatial concept using more natural utterances that do not specify a coordinate system. Furthermore, the proposed method demonstrated that it could automatically extract words representing concepts from unlabeled word sequences. We believe that this method can be extended to a more practical method that can learn concepts using utterances without word boundaries. The work can be beneficial for service robots to flexibly understand a new environment through the interactions with humans.

We intend to explore a method that improves the learning accuracy through modeling based on human recognition. First, we plan to adopt other coordinate systems. In this study, three coordinate systems were selected. However, in reality, humans may use other coordinate systems (Herskovits, 1986). Future work will involve accurate estimation of the coordinate system using a model closer to human recognition. Further, we plan to change the distribution of the spatial concepts. In the proposed method, normal distributions were used to express relative and absolute spatial concepts for facilitating analysis. However, in reality, most relative spatial concepts are related to the direction, such as “right” and “behind”, or the distance, such as “close to .ˮ. Concepts can be learned more accurately by expressing the distribution of the distance and angle. In addition, we plan to consider the size and shape of the object. In this study, we used the center point of the reference objects. However, when expressing a relative spatial concept using a large reference object, the represented range is wide. Therefore, learning will fail if reference objects of extremely different sizes are used. The same problem occurs when a long-or complex-shaped object is used. It is a future task to be able to learn relative spatial concepts considering the size and shape of the object. Furthermore, we plan to use extra-geometric relations to learn spatial concepts in addition to geometric relations. From the perspective of cognitive psychology, Coventry and Garrod argue that comprehension and production of spatial prepositions involves two types of constraints: geometric (spatial) constraints, and extra-geometric constraints (Coventry and Garrod, 2004). The teaching concepts in our task setting are affected by extra-geometric relations, e.g., object functions. It is a future task to improve the learning accuracy considering extra-geometric relations.

For an actual robot to learn a spatial concept using our method, it is necessary to eliminate the constraints further. For example, it is desirable to obtain the location and direction of an object from its environment. In particular, the direction of an object is highly ambiguous and difficult to learn. The object’s face, which defines the direction, can be estimated through supervised learning using the image and shape features of the object. In addition, it may be possible to learn the direction by simultaneous unsupervised learning of the object’s direction and spatial concepts, as well as those of the reference objects and coordinate systems.

## Figures and Tables

**FIGURE 5:**
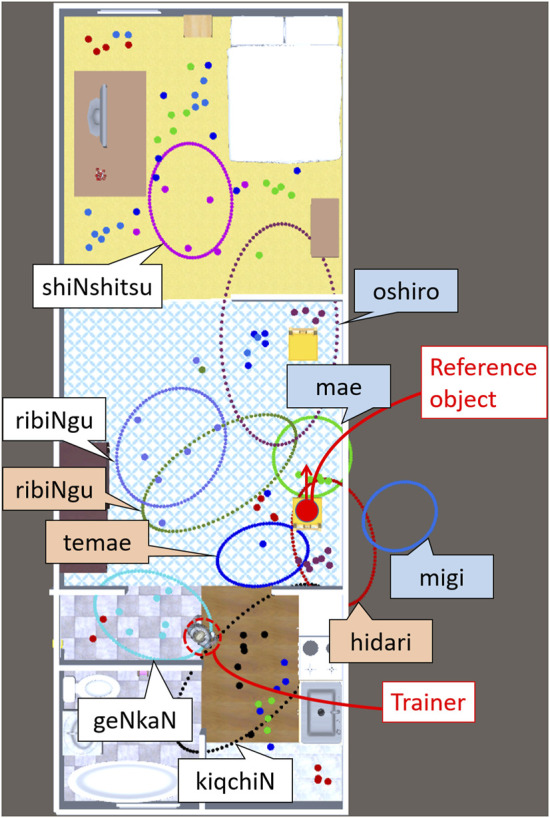
Locations taught by the trainer and spatial concepts learned in experiments with utterances. This is an example of the learning results using proposed method (D) without word boundaries. The word “ushiro” was recognized as the wrong phoneme sequence “oshiro.”

## Data Availability

The raw data supporting the conclusion of this article will be made available by the authors, without undue reservation.

## Footnotes

1 As all the experiments in this paper were performed in Japanese language, we provide English translations as well as Japanese phoneme sequences in this paper. The proposed method is applicable to languages other than Japanese.

2
https://github.com/a-taniguchi/SweetHome3D_rooms.

3 Word sequences were generated using the utterance patterns and location words shown in a Supplementary Material.

4 The robot can segment spoken utterances into word sequences to a certain degree using unsupervised word segmentation even if there are no words in language model the robot has. We show an experiment with spoken user utterances in Appendix A.

5 We performed Shapiro-Wilk tests for testing the normality of RAR, ARI, and WAR of the proposed method. The null hypothesis that the values were from normal distributions was rejected for RAR $\left(p=8.72\times 10^{-6}\right)$ , ARI $\left(p=1.22\times 10^{-4}\right)$ , and WAR $\left(p=1.22\times 10^{-4}\right)$ . Therefore, we performed Wilcoxon rank sum tests instead of Welch’s *t*-tests.

## Appendix A: Learning concepts using user utterances

We verified whether the proposed method could learn concepts using user utterances in which word boundaries were not obtained.

## A.1 Conditions

We used the utterances of the word sequences used in Section 5 spoken by a Japanese speaker. Julius 4.5 (Lee et al., 2001; Lee and Kawahara, 2009) was used for speech recognition. Only Japanese syllables were registered in the initial language model. Latticelm v0.4 was used for step (a) described in Section 4.2. The word segmentation results were obtained by skipping steps (b) and (c) and repeating steps (a) and (d) thrice. Examples of the obtained word sequences are shown in Table 5. There are segmentation errors such as “isu/no” becoming “isuno” and “geNkaN/niirune” becoming “gye/NkaNgiru/ne.” In addition, there are phoneme errors such as “beqdo” becoming “beqto.” In contrast, some are recognized as “beqdo” correctly. Thus, there are cases where one word is segmented into multiple types of words. To tolerate these phoneme recognition errors, a metric PAR defined as the average value of the phoneme accuracy rate of each utterance is used instead of metric WAR. We compared the result of the proposed method (D, E) when word boundaries were obtained (experiment in Section 5) and not obtained.

**TABLE 5:** Examples of the word segmentation results. The green and red words denote an object word and a location word, respectively.

Translation to English	Word sequences	Segmentation results
Here is in front of the bed	sokowa/beqdo/no/mae	sokowa/beqto/no/mae
You are left of the chair	isu/no/hidari/niimasu	isuno/hidari/ni/ma/su
You are in the entrance	geNkaN/niirune	gye/NkaNgiru/ne
Here is the bedroom	sokowa/shiNshitsu/dayo	sokowa/shiNshitsu/dayo

## A.2 Results

The experimental results are listed in Table 6. As expected, when either method was used, the result without word boundaries was lower than the result with word boundaries. The failure to learn word distributions owing to word segmentation errors causes inappropriate clustering of the locations. Figure 5 shows a learning example by method (D) whose evaluation values are close to the average: CAR = 0.731, RAR = 0.806, ARI = 0.750, PAR = 0.797. Some concepts are incorrectly learned. Concept “hidari” is erroneously learned as an egocentric coordinate system rather than an intrinsic coordinate system. In addition, concept “oku” is not learned. This is due to incorrect word segmentation because the word “oku” appears relatively infrequently. Word “oku” is divided into three types, “oku,” “nooku,” and “mooku.” This shows that the relationship between the concept and word is not learned correctly. The learning accuracy may be improved by increasing the variations in teaching. In addition, this can be solved by improving the word segmentation using the distribution of the spatial concept, such as steps (c) and (d) of ReSCAM+O. As we focus on learning the spatial concepts by selecting the coordinate system in this study, such an improvement is needs to be undertaken in future.

**TABLE 6:** Evaluation results using the proposed method.

Condition	Method	Results			
Word boundaries are obtained		CAR	RAR	ARI	PAR
✓	(D) $\lambda_{0}^{\text{R}}=0.01$	0.883	0.782	0.832	0.818
✓	(E) $\lambda_{0}^{\text{R}}=1.00$	0.964	0.984	0.945	0.964
	(D) $\lambda_{0}^{\text{R}}=0.01$	0.805	0.757	0.698	0.748
	(E) $\lambda_{0}^{\text{R}}=1.00$	0.787	0.963	0.723	0.773
