# Supplementary material for: Automatic selection of coordinate systems for learning relative and absolute spatial concepts
Source: Front Robot AI. 2022 Aug 12;9:904751. doi: 10.3389/frobt.2022.904751 (PMC9411740; doi:10.3389/frobt.2022.904751)
Supplement: Supplementary file 1 [file Table1.DOCX]

| TABLE 1 (A) Utterance patterns used in the experiments. In “***”, a phrase representing location is inserted. For example, “terebi no migi”, which means “right of the TV,” is inserted. (B)Location words used in the experiments.  **A**   \| Utterance patterns \| Translation to English \| \| --- \| --- \| \| *** dane \| It is ***. \| \| *** dayo \| It is ***. \| \| *** desu \| It is ***. \| \| *** niirune \| You are ***. \| \| *** niiruyo \| You are ***. \| \| *** niimasu \| You are ***. \| \| *** nikimashita \| You arrive ***. \| \| sokowa *** \| Here is ***. \| \| sokononamaewa *** \| The name of this place is ***. \| \| sonobashowa *** \| This place is ***. \| \| sokowa *** dane \| Here is ***. \| \| sokononamaewa *** dane \| The name of this place is ***. \| \| sonobashowa *** dane \| This place is ***. \| \| sokowa *** dayo \| Here is ***. \| \| sokononamaewa *** dayo \| The name of this place is ***. \| \| sonobashowa *** dayo \| This place is ***. \| \| sokowa *** desu \| Here is ***. \| \| sokononamaewa *** desu \| The name of this place is ***. \| \| sonobashowa *** desu \| This place is ***. \| |
| --- | --- | --- | --- | --- | --- | --- | --- | --- | --- | --- | --- | --- | --- | --- | --- | --- | --- | --- | --- | --- | --- | --- | --- | --- | --- | --- | --- | --- | --- | --- | --- | --- | --- | --- | --- | --- | --- | --- | --- | --- |
| **B**   \| Coordinate  system \| Word \| English \| \| --- \| --- \| --- \| \| Absolute \| kiqchiN \| kitchen \| \| geNkaN \| entrance \| \| shiNshitsu \| bedroom \| \| ribiNgu \| living room \| \| Intrinsic \| mae \| front \| \| ushiro \| back \| \| hidari \| left \| \| migi \| right \| \| Egocentric \| temae \| front (opposite side of “behind”) \| \| oku \| behind \| |
